# Serum and Lipoprotein Particle miRNA Profile in Uremia Patients

**DOI:** 10.3390/genes9110533

**Published:** 2018-11-05

**Authors:** Markus Axmann, Sabine M. Meier, Andreas Karner, Witta Strobl, Herbert Stangl, Birgit Plochberger

**Affiliations:** 1 Center for Pathobiochemistry and Genetics, Institute of Medical Chemistry and Pathobiochemistry, Medical University Vienna, 1090 Vienna, Austria; markus.axmann@meduniwien.ac.at (M.A.); sabine.meier@meduniwien.ac.at (S.M.M.); witta.strobl@meduniwien.ac.at (W.S.); 2 University of Applied Sciences Upper Austria, School of Medical Engineering and Applied Social Sciences, 4020 Linz, Austria; andreas.karner@fh-linz.at

**Keywords:** microRNA, lipoprotein, transfer, uremia

## Abstract

microRNAs (miRNAs) are post-transcriptional regulators of messenger RNA (mRNA), and transported through the whole organism by—but not limited to—lipoprotein particles. Here, we address the miRNA profile in serum and lipoprotein particles of healthy individuals in comparison with patients with uremia. Moreover, we quantitatively determined the cellular lipoprotein-particle-uptake dependence on the density of lipoprotein particle receptors and present a method for enhancement of the transfer efficiency. We observed a significant increase of the cellular miRNA level using reconstituted high-density lipoprotein (HDL) particles artificially loaded with miRNA, whereas incubation with native HDL particles yielded no measurable effect. Thus, we conclude that no relevant effect of lipoprotein-particle-mediated miRNA-transfer exists under in vivo conditions though the miRNA profile of lipoprotein particles can be used as a diagnostic marker.

## 1. Introduction

In general, intercellular communication occurs via cell-to-cell adhesion or by secretory messengers via e.g., hormones. A majority of circulating microRNAs (miRNAs) are derived from shedding of plasma membrane vesicles [1,2,3,4]. However, the protein Argonaute2 was also reported to carry a population of circulating miRNA without the need for vesicles [5]. Recently, high-density lipoprotein (HDL) particles were shown to transfer proteins and miRNAs from donor to recipient cells [6,7]. miRNAs are a class of small, non-coding RNAs which have emerged as important post-transcriptional regulators [8]. They intracellularly target specific messenger RNAs (mRNAs) to degrade or repress the translation process [8,9,10,11]. In extracellular space, miRNAs were thought to be relative unstable molecules; however, surprisingly, they were found to circulate in a highly stable form in various body fluids like blood, milk, saliva and urine [12]. Turchinovich et al. [13] proposed that extracellular miRNA carried by HDL is associated with the Argonaute family of proteins. However, proteome analysis did not reveal these proteins as a component of HDL [14,15,16,17,18,19]. The profile of circulating miRNAs display characteristic changes when pathogenic alterations occur. This dysregulation is linked to diseases such as cancer, cardiovascular [20,21,22,23,24] and kidney diseases [25,26,27,28]. Altered miRNA profiles in the HDL fraction of patients suffering from familial hypercholesterolemia were seen [6]. Thus, they may serve as markers for various diseases [29] and their progression [12] or the development of new therapeutic agents [8,30]. Thereby, mode and specificity of HDL uptake routes may influence the functional role of secreted miRNAs. This is one of the least understood issues in the field of miRNAs.

Here, we present data that miRNA is present—besides serum—in all lipoprotein particles and find that the miRNA profile is different in patients with uremia. Finally, we aimed to follow the uptake of miRNA to cells using quantitative real-time PCR (qPCR). For this purpose, we derived a protocol to increase the uptake for miRNA from HDL particles significantly. Taken together, our data indicate that—despite the presence of miRNA on lipoprotein particles—its abundance is rather low, which makes any influence on the cell metabolism via lipoprotein-mediated transfer in vivo generally highly unlikely.

## 2. Materials and Methods

The manuscript and the used nomenclature are written according to the minimum information for publication of quantitative real-time PCR (qPCR) experiments (MIQE) guidelines [31].

### 2.1. Patients

For the miRNA TaqMan™ (Applied Biosystems, Carlsbad, CA, USA) array analyses, 17 adult chronic renal failure (CRF) patients suffering from CRF Kidney Disease (Outcomes Quality Initiative stage 3–5 without hemodialysis) and 14 adult CRF patients on maintenance hemodialysis and matched controls were recruited (for lipid analyses see Table 1 of Meier et al. [32]). This study was approved by the Ethics Committee, Medical University of Vienna (EK-Nr. 511/2007). Written informed consent was obtained from all participants. Patients with an age over 55 years, with diabetes mellitus, nephrotic syndrome, severe hyperlipidemia, inflammatory diseases, malignancy and infections within the last three months, and patients on corticosteroids, lipid lowering or immunosuppressive drugs were excluded. Venous blood was collected after an overnight fast. Sera were frozen in aliquots at −20 °C. Lipoprotein particles were isolated immediately and stored frozen in aliquots at −20 °C until analysis. For detailed lipid and apolipoprotein analyses see Meier et al. [32].

### 2.2. microRNA in Serum and Lipoprotein Particles

As there is no known endogenous control for miRNAs in serum or lipoprotein particles, we added cel-miR-39 from the *C. elegans* species as internal control and for reference [33]. Total miRNA was reverse transcribed using the TaqMan™ microRNA Reverse Transcription Kit and the appropriate reverse transcription (RT) primers (Applied Biosystems), according to the manufacturer’s protocol. Resulting samples were subjected to RT-qPCR using TaqMan™ Gene Expression Universal Master Mix (Applied Biosystems) and TaqMan™ miRNA Assays (Applied Biosystems) following the manufacturer’s instructions. The amplification was conducted in a StepOne Real-Time PCR-System (48-well, Applied Biosystems); data was collected using the StepOne Software v2.1 (Applied Biosystems).

### 2.3. TaqMan™ Arrays

Equal volumes of patient samples were pooled and miRNA was isolated from 100 µL serum or 500 µg lipoprotein particles using the miRNeasy Kit (QIAGEN GmbH, Hilden, Germany), according to the supplier’s instructions, in two independent experiments. RNA quantity and purity was measured using a NanoDrop ND-1000 Spectrophotometer (peqlab Biotechnologie GmbH, Erlangen, Germany) and RNA was stored at −80 °C. Total miRNA was reverse transcribed using the TaqMan™ microRNA RT Kit with MegaPlex RT Primers (Applied Biosystems). To ensure the sample content for the TaqMan™ arrays, miRNAs from lipoprotein particles and serum were further processed by applying preamplification using the appropriate primers and the PreAmp Master Mix (Applied Biosystems). Afterwards, samples were loaded into TaqMan™ Array Cards A+B (in total 754 miRNAs were detected) and analyzed using a 7900HT Fast Real-Time PCR System (Applied Biosystems). Data was collected and analyzed using appropriate software from Applied Biosystems (SDS 2.4 and Data assist v3.0) yielding ΔΔc_q_. The value RQ (relative quantification) is defined as $RQ=2^{-\Delta \Delta c_{q}}$.

### 2.4. Synthetic miRNA

Human mature miRNAs hsa-miR145 (5′-GUC CAG UUU UCC CAG GAA UCC CU-3′), hsa-miR155 (5′-UUA AUG CUA AUC GUC AUA GGG GU-3′) and hsa-miR223 (5′-UGU CAG UUU GUC AAA UAC CCC A-3′) were synthesized by Microsynth (Microsynth, Vienna, Austria). The manufacturer did purification with HPLC & dialysis. miRNAs were solubilized in 10 mM tris(hydroxymethyl) aminomethane (TRIS) buffer, pH 7.5 (Thermo Fisher Scientific, Vienna, Austria) and stored at −20 °C in aliquots of 100 µL (final storage concentration 10 µM).

### 2.5. Lipoprotein Particle Isolation

For the reconstitution/labeling experiments, human plasma was collected from two normolipidemic healthy volunteers twice (time between donations was roughly one month), in accordance with the medical and ethical guidelines of the Medical University of Vienna. This part of the study was approved by the Ethics Committee, Medical University of Vienna (EK-Nr. 1414/2016). Written informed consent was obtained from all participants. Individual lipoprotein particle (HDL and low-density lipoprotein (LDL)) fractions were isolated by serial ultra-centrifugation at a density of 1.21 g/mL or 1.06 g/mL, respectively [34]. Final protein concentration was determined photometrically (Bradford assay) and samples were stored under an inert atmosphere at +4 °C. For the preparation of lipoprotein particle deficient serum (LPDS), human sera from both donors were spun using ultra-centrifugation at a density of 1.21 g/mL, dialyzed and stored at −20 °C.

### 2.6. Reconstitution of HDL Particles

HDL particles were reconstituted by a modified protocol, previously published in [35]. In short, lipids from HDL particles were extracted two times with ethanol : diethyl ether (3:2) at −20 °C for 2 h. Precipitate was dried under nitrogen gas flow and resuspended in buffer A (150 mM NaCl, 0.1‰ ethylenediaminetetraacetic acid (EDTA), 10 mM TRIS/HCl, pH 8.0, all Sigma Aldrich, Vienna, Austria). Protein concentration was determined photometrically (Bradford assay). A lipid mixture, consisting of l-α-phosphatidylcholine, cholesterol oleate and cholesterol (all Sigma Aldrich) at a molar ratio of 100:22:4.8 dissolved in chloroform : methanol (2:1), was dried under nitrogen gas and resuspended in buffer A. Aliquots of synthetic miRNAs (100 µL, 10 µM) were mixed with freshly prepared spermine solution (final concentration 15 mM, Sigma Aldrich) for 30 min at 30 °C. Lipid suspension and miRNA/spermine solution were mixed and sodium deoxycholate (Sigma Aldrich) was added for lipid solubilization at a final concentration of 15 mM. In negative control experiments, HDL particles were reconstituted without addition of miRNA and/or spermine. The mixture was stirred at +4 °C for 2 h. Delipidated HDL was added at a final molar ratio of l-α-phosphatidylcholine, cholesterol oleate, cholesterol and protein (HDL) of 100:22:4.8:1, and the mixture was stirred at +4 °C overnight. Extensive dialysis using Slide-A-Lyzer™ dialysis cassettes (cut-off 20 kDa, Thermo Fisher Scientific) against phosphate-buffered saline (PBS, Roth, Graz, Austria) + Amberlite XAD-2 polymeric adsorbent (15 g/L, Sigma Aldrich) was performed to separate generated reconstituted HDL (rHDL) particles form free miRNA, spermine and detergent. Final protein concentration was determined photometrically (Bradford assay) and samples were stored under inert atmosphere at +4 °C.

### 2.7. Labeling of LDL Particles

LDL particles were labeled with miRNA by a modified protocol, previously published in [36]. In short, LDL particle solution was incubated with 0.3 mM EDTA/0.1 mM ethylene glycol-bis(β-aminoethyl ether)-*N*,*N*,*N′*,*N′*-tetraacetic acid (EGTA, Roth) for 10 min at +4 °C. Aliquots of miRNA were mixed with freshly prepared spermine solution (final concentration 15 mM, Sigma Aldrich) for 30 min at 30 °C. After addition of an equal volume of dimethyl sulfoxide DMSO (Thermo Fisher Scientific), the mixture was diluted five-fold with buffer containing 150 mM NaCl/0.3 mM EDTA/0.1 mM EGTA. LDL particle solution and miRNA mixture were combined and incubated for 2 hours at 40 °C. In negative control experiments, LDL particles were labeled without addition of miRNA and/or spermine. Extensive dialysis using Slide-A-Lyzer™ dialysis cassettes (cut-off 20 kDa, Thermo Fisher Scientific) against PBS (Roth) + Amberlite XAD-2 polymeric adsorbent (15 g/L, Sigma Aldrich) was performed to separate labeled LDL particles from free miRNA, spermine and organic solvent. Final protein concentration was determined photometrically (Bradford assay) and samples were stored under inert atmosphere at +4 °C.

### 2.8. Quality Control of Reconstituted/Labeled Lipoprotein Particles

**Figure A1:**
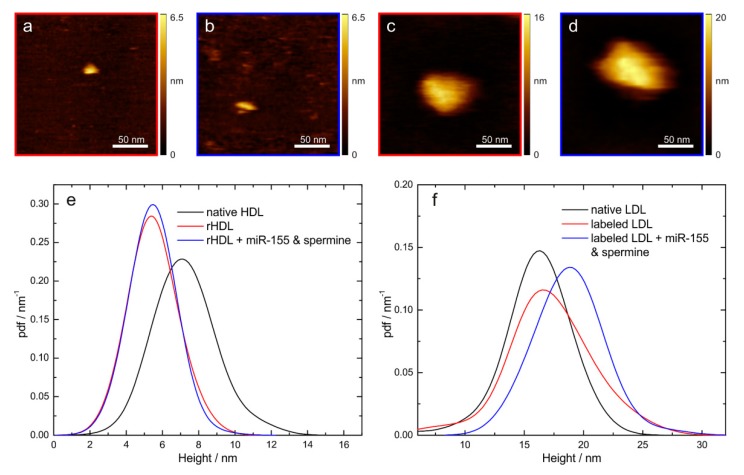
Spatial characterization of reconstituted/labeled lipoprotein particles. High-speed atomic force microscopy (HS-AFM) images of reconstituted HDL particles (**a**), rHDL particles with miR-155 & spermine (**b**), labeled LDL particles (**c**) and labeled LDL particles with miR-155 & spermine (**d**). The size analysis yielded distributions of particle heights for native HDL particles (N = 125 particles, <height> = 7.3 nm ± 1.5 nm), rHDL particles (N =110, <height> = 5.5 nm ± 1.0 nm) and rHDL particles + miR-155 & spermine (N = 122, <height> = 5.5 nm ± 0.9 nm) (**e**), as well as for native LDL particles (N = 79, <height> = 16.1 nm ± 2.4 nm), labeled LDL particles (N = 89, <height> = 17.2 nm ± 3.4 nm) and LDL particles + miR-155 & spermine (N = 57, <height> = 18.8 nm ± 2.4 nm) (**f**).

High-speed atomic force microscopy (HS-AFM) (see Figure A1) was performed in tapping mode with free amplitudes of 1.5 nm–2.5 nm. The amplitude setpoint was larger than 90% of the free oscillation amplitude. USC-F1.2-k0.15 cantilevers (Nanoworld AG, Neuchâtel, Switzerland) were used. HDL and LDL particle aliquots were diluted 1:10^3^ in PBS (in order to observe individual particles) and incubated for 5 min on freshly cleaved mica. After rinsing with PBS, the samples were imaged in PBS at room temperature. Image processing and particle analysis were done in Gwyddion 2.49 (CMI, Brno, Czech Republic) and SPIP (Image Metrology, Hørsholm, Denmark). The probability density functions (pdfs) representing the height distribution of particles were calculated using the ‘ks-density’ algorithm in MATLAB (Mathworks, Natick, MA, USA).

### 2.9. Cells

Chinese hamster ovary (CHO) cell lines CHOK1, ldlA7 (reduced expression of LDL-receptor), and ldlA7-SR-B1 (overexpression of scavenger receptor class B type 1 (SR-B1)) were cultured as previously described in [37,38]. In short, cells were maintained in Dulbecco’s modified Eagle Medium/Nutrient Mixture F-12 (Sigma Aldrich) supplemented with 10% fetal calf serum (FCS, Sigma Aldrich), Penicillin/Streptomycin (100 U/mL and 0.1 mg/mL final concentration, Sigma Aldrich) and 2 mM L-Glutamine (Roth). Geneticin sulphate (Thermo Fisher Scientific) was added to ldlA7-SR-B1 cells at a final concentration of 0.5 mg/mL as selection antibiotic.

Cells were grown until just reaching confluency in individual LabTek™ chambers (eight chambers per slide, each with a 4 mm × 4 mm surface area and ~700 µL total volume, Thermo Fisher Scientific). Cells were washed three times with Hanks’ balanced salt solution (HBSS, Roth) and incubated in FCS-free culture medium. Next, HDL particle solution (final concentration 50 µg/mL or 5 µg/mL) or LDL particle solution (final concentration 5 µg/mL) from the different preparations (native/reconstituted/labeled) was added. Incubation was performed at 37 °C and 5% CO_2_. After 16 h, cells were washed three times with HBSS and covered with 100 µL of medium without FCS. For negative control experiments, chambers without cells and with cells without any addition of particles were used. For blocking experiments (testing of unspecific binding), native lipoprotein particle solution or bovine serum albumin (BSA, Sigma Aldrich) was used. Cell number was determined for each experiment in two independent chambers, which were similarly treated as above (medium containing 50 µg/mL HDL or 5 µg/mL LDL particle solution) and the average cell number of both chambers was used for normalization.

### 2.10. miRNA-Extraction

miRNA-extraction was performed according to the manufacturer’s protocol of the miRNeasy Mini Kit (Qiagen, Vienna, Austria). In short, a sample volume (ranging from 1 µL to 100 µL (lowest protein concentration)) of native/reconstituted HDL particles or native/labeled LDL particles normalized to the lowest protein concentration was lysed and homogenized. The cell sample volume used for miRNA-extraction contained the pooled cells from two independent chambers. For the negative control sample, 100 µL of RNase-free water (Roth) was used. A phenol/chloroform extraction was performed subsequently to separate proteins/DNA from RNA.

### 2.11. Reverse Transcription

Reverse transcription was performed according to the manufacturer’s protocol of the TaqMan™ microRNA Reverse Transcription Kit (Thermo Fisher Scientific). Briefly, 7 µL of the kit’s mastermix solution were added to 5 µL of the extracted miRNA-sample and 3 µL of miRNA-specific primer (Assay ID 002623, 000526, and 002278, Thermo Fisher Scientific). In order to use the same cell number for each cell line, the sample volume of the cell line with a higher overall cell number was reduced accordingly; the residual volume to reach the total sample volume of 5 µL was RNase-free water (thus, sample volume from the individual cell lines varied between 5 µL and 2 µL). As a sample for standard curve preparation, an aliquot of miRNA was diluted sequentially in RNase-free water. Reverse transcription was performed using a Thermocycler (LabCycler Sensoquest, Göttingen, Germany) with the following program: (1) 30 min at 16 °C, (2) 30 min at 42 °C, (3) 5 min at 85 °C, (4) ∞ at 4 °C. Usually, the qPCR step was done immediately after the reverse transcription—otherwise the complementary DNA synthesized from the miRNA samples was stored at −20 °C.

### 2.12. qPCR

qPCR of the reverse transcribed samples was performed according to the manufacturer’s protocol of the TaqMan™ assay (Thermo Fisher Scientific). In short, per sample, 7.5 µL of mastermix solution (iTaq™, Biorad, Vienna, Austria) was diluted with 4.75 µL RNase-free water and 0.756 µL hydrolysis probe (TaqMan™ Assay ID 002623, 000526, and 002278, Thermo Fisher Scientific). Each sample contained further 2 µL of cDNA sample or RNase-free water and was measured twice. qPCR was performed using a Corbett RG-6000 PCR machine (Corbett Research, Cambridge, UK) with the following program: (1) 2 min at 50 °C, (2) 10 min at 95 °C, (3) 15 s at 95 °C, (4) 60 s at 60 °C. Steps (3) and (4) were repeated up to 50 times. For the analysis, RotorGene 6000 Software Version 1.7 (Qiagen, Vienna, Austria) was used with activated ‘DynamicTube Normalization’ (for compensation of different background levels using the second derivative of each sample trace) and ‘Noise Slope Correction’ (normalization to the noise level). The threshold for calculation of the quantification cycle c_q_ for each miRNA was determined from the standard curves of each miRNA individually, via the ‘Auto-Find Threshold’ function of the software package—hereby, the software maximizes the R-value of the fit of the standard curve—and kept equal for each specific miRNA. The c_q_ value of the negative control was always at least six cycles higher than the highest sample c_q_ value (in the range between 30–40 cycles). In each run, at least one of the standard dilutions of miRNA was added as a sample to calibrate each individual run for the same reaction efficiency.

### 2.13. Calculation of miRNA Strands Number

**Figure A2:**
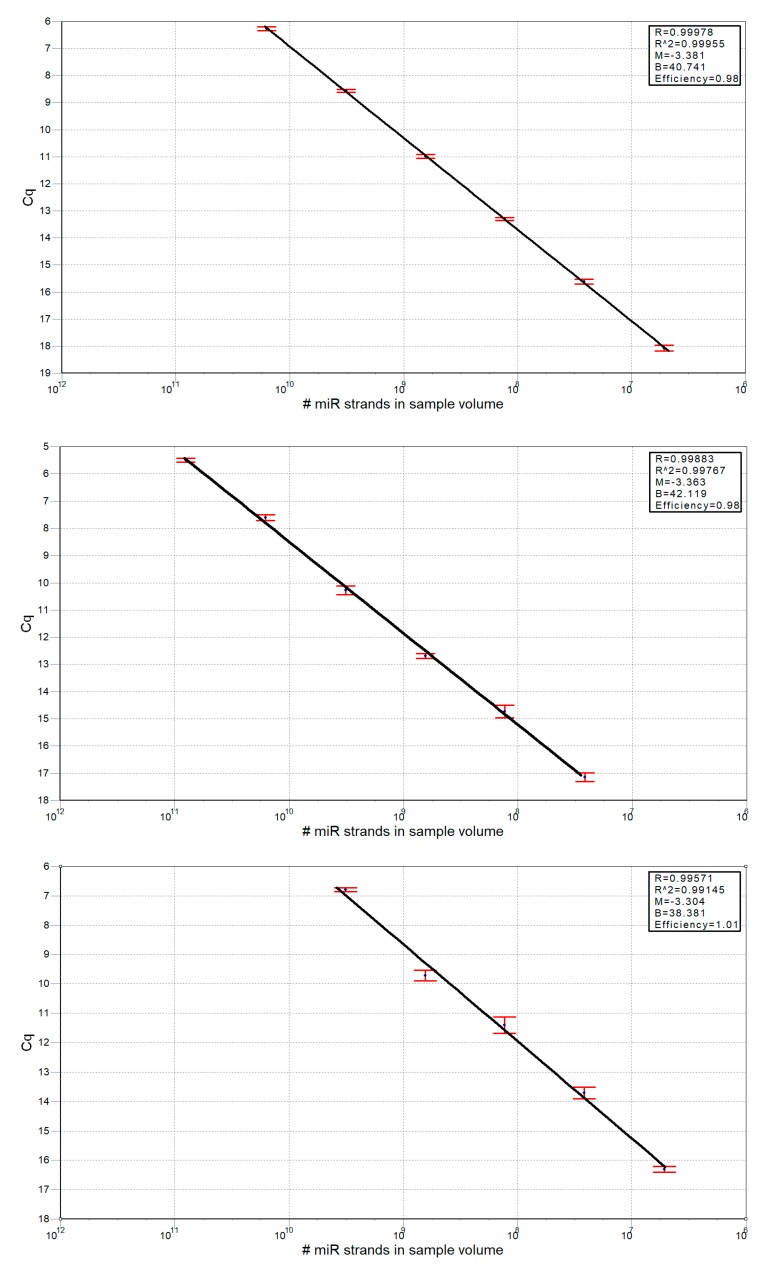
Standard curves for miR-145 (top), miR-155 (center) and miR-223 (bottom).

For standard curves (Figure A2), the number of miRNA strands of each sequential dilution step was calculated from the initial concentration and the subsequent dilution steps during reverse transcription and PCR. Thereby, we assumed that the reverse transcription performed linearly over the low concentration range used here and in a 1:1 stoichiometry. Accordingly, the number of strands corresponding to the c_q_ value of each sample run was calculated from the standard curve regression line and normalized to the number of cells or the lipoprotein particle number, respectively. Lipoprotein particle number was estimated from the initial protein concentration and its average molecular weight (molecular weight MW_HDL_ ~250 kDa, MW_LDL_ ~3 MDa). We assumed no lipid contribution to the molecular weight—thus, we slightly overestimated the number of miRNA strands per lipoprotein particle. We further assumed a 100% recovery of miRNA during the miRNA extraction step.

## 3. Results

### 3.1. miRNA in Lipoprotein Particle Fractions

First, we tested the presence of miRNA in serum and all lipoprotein particle fractions of healthy volunteers. To do so, we separated serum and lipoprotein particle fractions and purified the contained miRNAs. For analysis, we selected two miRNAs known to be involved in renal and cardiovascular disease, namely miR-21 [39] and miR-223 [40]. Both miRNAs were detectable in all fractions (Table 1) with the highest relative amount in HDL particles; however, the abundance was low (absolute c_q_ values varied between 26 and 36 cycles). This data indicates a lipoprotein-particle-selective association of miRNAs. To demonstrate that HDL particles did not contain remaining serum constituents, we diluted the HDL particle fraction with excess PBS and centrifuged it a second time. Still, this fraction contained the highest miRNA amount among the lipoprotein particle classes (<ΔΔc_q_ > = −2.5 (SD = 0.2) and −1.5 (SD = 0.1) for miR-223 and miR-21, respectively), indicating that miRNAs are relatively enriched at least in the HDL particle fraction.

**Figure 1:**
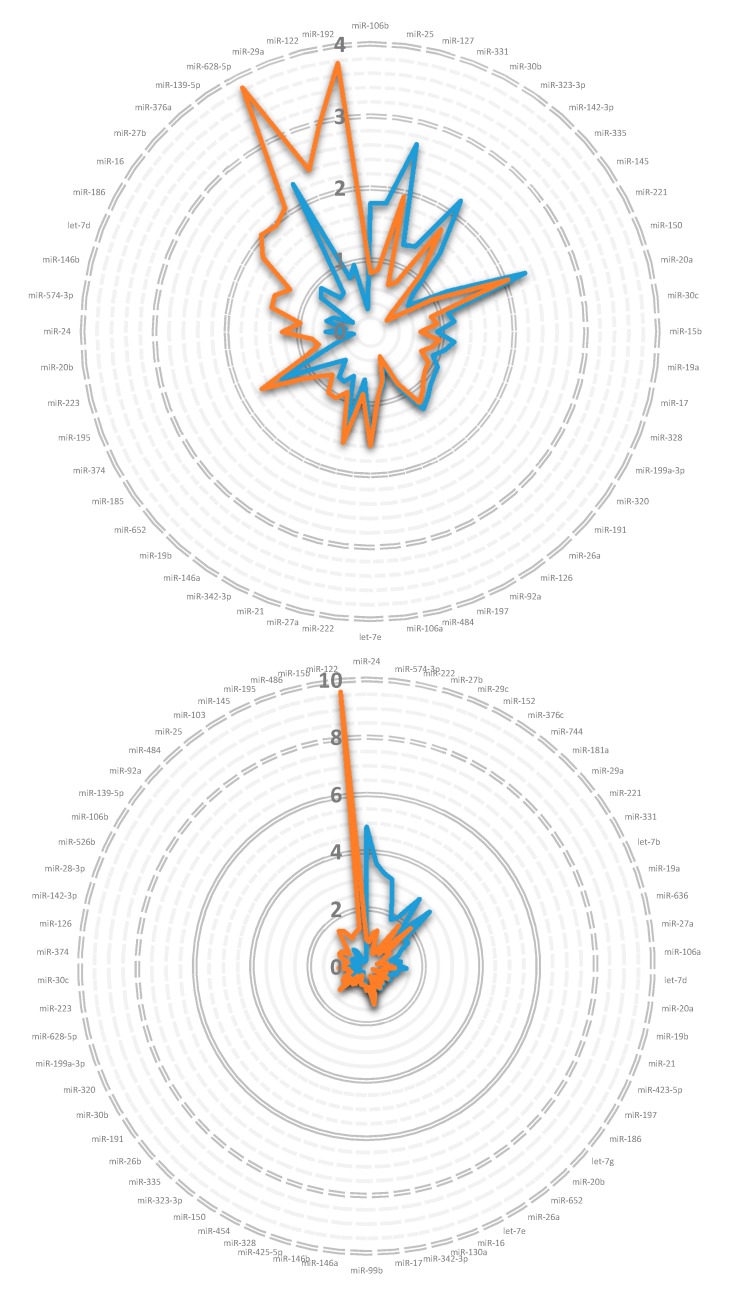
miRNA profile in serum and high-density lipoprotein (HDL) particles of chronic renal failure (CRF) and hemodialysis patients. Pooled serum and HDL particles of adult CRF (top) and hemodialysis patients (bottom) and their matched controls was analyzed by the TaqMan™ miRNA array A (duplicates). From the 381 different miRNAs, 57 (serum) and 90 (HDL particles) for CRF patients and 75 (serum) and 89 (HDL particles) for hemodialysis patients were detectable (plotted were only miRNAs detectable in serum and HDL particles) and the relative quantitation (RQ) was calculated subsequently (the corresponding control miRNAs were set to RQ = 1). RQ-values were arranged in the following manner: starting at the twelve o’clock position with the maximum negative difference between the RQ values of HDL particles (orange) and serum (blue), the values increase clockwise (reaching nearly equality at the six o’clock position) and reach the maximum positive difference before closing the circle again (see Table A2 (in Appendix A) for the complete data set).

Next, we investigated miRNA profiles in serum and HDL particles of uremic patients recruited for a study on the influence of chronic renal failure (CRF) on cholesterol efflux from macrophages [32]. Figure 1 depicts the expression profile of miRNAs found in the serum/HDL particles of patients suffering from CRF (top) and from the more advanced disease course (hemodialysis, bottom) relative to their matched healthy controls. In both settings, the miRNA profile difference between serum and HDL particles was diverse: some miRNAs were represented in HDL particles and serum in a similar portion (see Figure 1 overlap of the blue and orange line); however, some miRNAs were increased at different rates in serum and HDL particles. The most regulated miRNA in CRF patients—miR-192—was about 3.8 times increased in HDL particles while it was decreased in serum to one-third. Nevertheless, there was a substantial overlap in the miRNA signature between serum and HDL particles in both CRF and hemodialysis patients.

**Figure 2:**
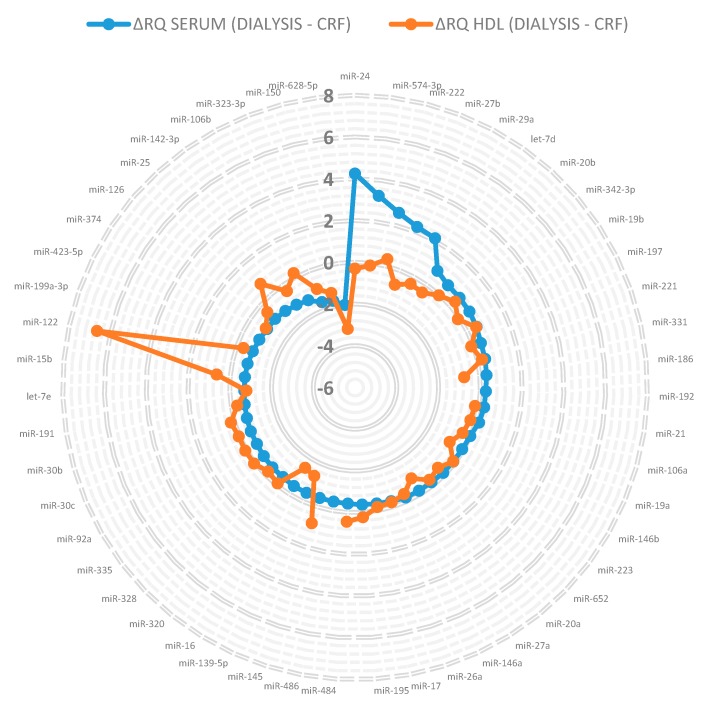
Differences in the miRNA profile in serum and HDL particles during disease progression from CRF to hemodialysis. Data is arranged in the following manner: starting at the twelve o’clock position with the maximum difference between the RQ values of serum (blue) during disease progression from CRF to hemodialysis (=ΔRQ), the values decrease clockwise (reaching nearly no change at the four o’clock position) and reach the maximum negative difference before closing the circle again (plotted are only miRNAs detectable in both disease progression steps). For comparison, ΔRQ values were superimposed for the same miRNAs (if available) in the HDL particle fraction (orange).

Furthermore, we assessed the differences in the serum and HDL signatures between the two patient cohorts; most of the changes in the miRNA levels were in a similar direction (Figure 2). The miR-122 level increased sharply in the HDL fraction with disease progression (ΔRQ ~ +6.6), while it hardly decreased in the serum (ΔRQ ~ −0.7). On the other hand, some miRNA levels like miR-24, miR574-3p, miR-222, miR-27b and miR-29a were increased in the serum with disease progression, whereas in HDL particles, the levels remained nearly constant. While these miRNA levels were significantly different between HDL particles and serum, a substantial overlap in the miRNA profile exists.

### 3.2. miRNA Associated to Lipoprotein Particles

After having shown that miRNA also circulates with lipoprotein particles and that their expression profile was altered in the disease progression, we assessed if we can reconstitute this system in order to observe and follow cellular miRNA uptake. Therefore, we tested the uptake of native HDL/LDL and reconstituted HDL/labeled LDL particles with two selected miRNAs: miR-223, which shows a high abundance and miR-155, which is rare in lipoprotein particles [41]. To assess the validity of the reconstituted and labeled lipoprotein particles in comparison to their native counterpart, we imaged single lipoprotein particles by HS-AFM and analyzed their size (Figure A1). We observed globular particles comparable to the native forms. However, the reconstituted HDL particle variants were slightly smaller than the native ones; reasonably due to the partial reconstitution process. For LDL particles, the size distributions of the native and labeled particles largely overlap; obviously, spermine addition accounts for the observed size increase. According to our data, the ratio of tested miRNA strands to the number of native lipoprotein particles was in the range of one strand of a specific miRNA to around 1 billion lipoprotein particles (Table 2 and Table 3).

Nevertheless, we were able to increase the miRNA/particle ratio via reconstitution or labeling of the respective lipoprotein particle. The reconstitution (which involves thorough delipidation of the particle) or the labeling process of the lipoprotein particle itself did not influence the miRNA/particle ratio (compare miRNA/particle ratio of native and reconstituted/labeled particles). Thus, we found neither sequence-specificity (no significant difference between miRNA/particle ratio of miR-223 and miR-155), nor any significant effect of electrical-charge-compensation of the poly-anionic nature of the miRNA with addition of the natural poly-cationic nucleotide-sequence-stabilizer-agent spermine. However, our approach increased the miRNA/particle ratio by around five orders of magnitude (from 10^−9^ to 10^−4^ for HDL particles and 10^−8^ to 10^−3^ for LDL particles as shown in Table 2 and Table 3).

Next, we tested whether the uptake of lipoprotein particles in cells depends on the cell membrane density of their respective lipoprotein receptor. In order to reach a steady state between uptake and potential degradation of miRNA, we incubated cells for 16 h with lipoprotein particles. We observed no increase of the cellular miRNA-content after incubation with native lipoprotein particles at a concentration in the physiological range (5 µg/mL and 50 µg/mL for HDL or LDL particles) independent of the receptor density (Table 4 and Table 5). We did neither observe a significant increase in the number of miRNA strands per cell for HDL particles (overexpression of the HDL-receptor SR-B1 in ldlA7-SR-B1 cells in comparison with native HDL-receptor density in ldlA7 cells) nor for LDL particles (a low LDL-receptor density in ldlA7 cells in comparison with native LDL-receptor density in CHOK1).

However, incubation using lipoprotein particles with artificially increased miRNA content yielded—at least for HDL particles—a significant and receptor-density-dependent increase of the number of miRNA strands per cell (approximately by a factor 4 or 10 at native or overexpressed receptor densities). In contrast, incubation with LDL particles did not influence the miRNA level.

In order to exclude the possibility of a non-cell-mediated unspecific interaction of HDL particles with the cell culture vessel itself, we performed control experiments with chambers without cells (see Table A1). Here, the determined numbers for the miRNA amount corresponding to an individual cell (comparable to numbers from Table 4 and Table 5) were around 1% and were, therefore, neglected. An incubation-concentration-dependence of the unspecific binding could be seen, and it was further observed that pre-blockage with BSA or native HDL particles had no influence.

## 4. Discussion

We have shown that, for some miRNAs, there is a significant difference in the profile between the HDL particle fraction and serum derived from CRF as well as hemodialysis patients. For other miRNAs, their values are nearly identical. Moreover, during disease progression, a similar bimodal behavior was observable. Especially miR-24, which has been reported to be upregulated in patients with kidney transplants [42], showed an increase of more than four RQ values in the serum, while its value even decreased in the HDL particle fraction by more than three values. Contrarily, RQ values of miR-122 increased by more than six values during disease progression in the HDL particle fraction while the serum RQ value hardly decreased. Interestingly, miR-21 and miR-223—two miRNAs known to be involved in renal and cardiovascular disease—did not change, neither in serum nor in the HDL particle fraction. Thus, we postulate that the miRNA profile may be suitable to identify certain diseases and follow their progression [43]; however, one has to be careful regarding its origin, as some miRNA levels differ between serum and HDL particle fraction.

Vickers et al. showed that HDL particles deliver miR-223 to SR-B1–transfected baby hamster kidney (BHK) cells, leading to repression of the Renilla-SR-B1-3′UTR luciferase reporters [6]. Therefore, HDL particles can be expected to transport miRNAs to target cells, leading to altered gene expression. Tabet et al. demonstrated that HDL-transported miR-223 down-regulated ICAM-1 expression in endothelial cells [7]. In the present study, we verified the principal functionality of HDL particles as miRNA-transfer-vehicle. Nevertheless, the number of miRNAs transported via native HDL particles may be insignificant due to the extremely low miRNA/particle ratio. This number may slightly vary depending on the miRNA and individual; however, any relevant influence on the cellular mRNA profile due to an uptake of miRNA-containing lipoprotein particles seems highly unlikely—at least in normolipidemic healthy individuals. We like to note that our numbers (strands/particle) are comparable to data published by Dimmler et al. (10^4^ strands/µg lipoproteins are comparable to 10^−9^ strands/particle) [41].

Moreover, we have estimated that for the observed increase of miRNA strands per cell at standard receptor density, each cell has to interact, on average, with at least 7.5 million HDL particles (assuming a miRNA/particle ratio of 1:10^4^, see Table 1) during the incubation period of 16 hours. Under the assumption of no intracellular degradation of miRNA, this value represents only a lower limit. This yields a minimum of roughly 60 uptake events of HDL particles per second—a calculation previously unavailable, which enables a rough estimation of the absolute cargo transfer. According to our measured ratio of 10^−9^ miRNA strands per native HDL particle, on average, it would take more than half a year until a single miRNA strand has been taken up by a single cell. In contrast to HDL-mediated miRNA-uptake, we did not observe a significant increase of the miRNA level using LDL particles—an observation in agreement with the different lysosomal uptake pathway of LDL, which leads to degradation of the cargo itself.

Furthermore, our observation that reconstitution and the associated delipidation step did not influence the miRNA/particle ratio leads us to speculate that miRNA itself is not dominantly lipid-associated, and thus, not associated to the particle surface. Experiments using fluorescently labeled miRNA added to planar supported lipid bilayers (data not shown) yielded no observable signal—independent of the tested conditions (lipid composition, miRNA-concentration, temperature, pH-value, label). Moreover, extra-particular electrostatic association seems unlikely, due to the negative surface potential of lipoprotein particles and the poly-anionic nature of RNA strands. In addition, miRNAs are usually incorporated into a protein of the Argonaute family (AGO1-4) and have been proposed to circulate with HDL [13]. More than 150 proteins were found within the HDL particle fraction using proteome analysis; 95 of these proteins were identified to be specifically bound to the HDL particle. However, neither of the AGO proteins were detected [14,15,16,17,18,19]. Additionally, using Western Blot analysis and fluorescence imaging, we were unable to detect any AGO-2 protein (unpublished data). Therefore, it seems to be unlikely that this protein family is carrying the miRNA of HDL particles. The protein moiety enabling some of the HDL particles to bind miRNA still remains elusive.

In summary, we conclude that miRNA is transported and transferred to cells via lipoprotein particles, however, most likely plays no relevant role in vivo regarding the alteration of the cellular miRNA, and thus, the mRNA profile. However, lipoprotein particles may serve as diagnostic tools for certain disease-specific miRNAs—as exemplarily demonstrated here by us—or moreover, as an artificial drug delivery system.

## Figures and Tables

**Table 1:** miRNAs circulating in human serum and associated to lipoprotein particles.

Sample	miRNA	< ΔΔc_q_ >	SD < ΔΔc_q_ >
LPDS	223	0.0	0.1
	21	0.0	0.2
VLDL	223	5.7	0.2
	21	4.6	0.2
LDL	223	2.8	0.2
	21	1.8	0.2
HDL	223	−3.5	0.1
	21	−2.5	0.1

Lipoprotein particle deficient serum (LPDS) and lipoprotein particle fractions (very-low density lipoprotein (VLDL), low density lipoprotein (LDL) and high density lipoprotein (HDL) particles) were analyzed for two distinct microRNAs (miRNAs). Values are in relation to the levels of cel-miR-39 and subsequently LPDS. (< x > = average value of x, SD = standard deviation, n = 3 independent experiments).

**Table 2:** * miRNA content of HDL particle variants.

Sample	Donor	miRNA	< # miRNA/particle >	SD < # miRNA/particle >
Native HDL	1	223	6.10^−9^	7.10^−10^
		155	3.10^−9^	9.10^−10^
	2	223	7.10^−9^	4.10^−9^
		155	4.10^−8^	2.10^−8^
rHDL	1	223	4.10^−9^	8.10^−10^
		155	5.10^−9^	3.10^−9^
	2	223	7.10^−9^	4.10^−9^
		155	1.10^−8^	1.10^−8^
rHDL + miR-155	1	155	1.10^−4^	1.10^−4^
	2	155	8.10^−5^	5.10^−5^
rHDL + miR-155 & spermine	1	155	1.10^−4^	5.10^−5^
	2	155	7.10^−5^	2.10^−5^

* Each condition was tested twice with two independently obtained samples. rHDL: reconstituted HDL.

**Table 3:** * miRNA content of LDL particle variants.

Sample	Donor	miRNA	< # miRNA/particle >	SD < # miRNA/particle >
Native LDL	1	223	3.10^−8^	2.10^−8^
		155	9.10^−8^	5.10^−8^
	2	223	5.10^−9^	3.10^−9^
		155	9.10^−8^	2.10^−8^
Labeled LDL	1	223	5.10^−8^	3.10^−8^
		155	9.10^−8^	5.10^−8^
	2	223	4.10^−9^	3.10^−9^
		155	9.10^−8^	2.10^−8^
Labeled LDL + miR-155	1	155	1.10^−4^	6.10^−5^
	2	155	2.10^−3^	3.10^−4^
Labeled LDL + miR-155 & spermine	1	155	5.10^−4^	3.10^−4^
	2	155	5.10^−3^	6.10^−4^

* Each condition was tested twice with two independently obtained samples.

**Table 4:** * Influence of lipoprotein receptor density on cellular miRNA-content before/after HDL particle uptake.

Cell Line	HDL Conditions and Concentration	< # miRNA/cell >	SD < # miRNA/cell >
ldlA7	no HDL addition	240	35
	native, 50 µg/mL	250	25
	miR-155 and spermine, 50 µg/mL	980	55
ldlA7-SR-B1	no HDL addition	260	20
	native, 50 µg/mL	330	55
	miR-155 and spermine, 50 µg/mL	2500	190

* Each condition was tested twice with two independently obtained samples. The HDL particles with the highest < # miRNA/particle > = 1 × 10^−4^ were used for these experiments. The used sample volume in the qPCR step equated for both cell lines to 3100 cells.

**Table 5:** * Influence of lipoprotein particle receptor density on cellular miRNA-content before/after LDL particle uptake.

Cell Line	LDL Conditions and Concentration	< # miR/cell >	SD < # miR/cell >
ldlA7	no LDL addition	190	10
	native, 5 µg/mL	240	20
	miR-155 and spermine, 5 µg/mL	210	40
CHOK1	no LDL addition	120	10
	native, 5 µg/mL	130	20
	miR-155 and spermine, 5 µg/mL	160	70

* Each condition was tested twice with two independently obtained samples. The LDL particles with the highest < # miRNA/particle > = 5 × 10^−3^ were used for these experiments. The used sample volume in the qPCR step equated for both cell lines to 5800 cells.

## Appendix A

**Table A1:** Unspecific binding of HDL particles to the chamber.

Chamber Conditions	< # miRNA >	SD < # miRNA >
Blocking with 5% BSA for 1 h + medium for 16 h	2600	5
Blocking with 500 µg/mL native HDL for 1 h + medium for 16 h	2200	90
Blocking with 5% BSA for 1 h + 50 µg/mL rHDL + miR-155 & spermine for 16 h	14500	2800
Blocking with 500 µg/mL native HDL for 1 h + 50 µg/mL rHDL + miR-155 & spermine for 16 h	11000	2800
50 µg/mL rHDL + miR-155 & spermine for 16 h	11000	2100
5 µg/mL rHDL + miR-155 & spermine for 16 h	2800	140

The average number of miRNA < # miRNA > corresponds to the same volume as is contained in the chamber with ldlA7-SR-B1 cells (Table 4). For the authors, the only reasonable way to compare these values to the numbers from Table 4 is to divide them by the number of cells used (there ~3100 cells).

**Table A2:** RQ Values from Figure 1 of CRF (top) and hemodialysis patients (bottom).

Assay	RQ_SERUM_	RQ_HDL_	Assay	RQ_SERUM_	RQ_HDL_	Assay	RQ_SERUM_	RQ_HDL_
miR-106b	1.78	0.82	**miR-19b**	0.52	0.80	**miR-425-5p**	N.D.	0.83
miR-25	1.80	0.87	**miR-652**	0.64	0.93	**miR-148b**	N.D.	0.85
miR-127	2.68	1.94	**miR-185**	0.86	1.16	**miR-485-3p**	N.D.	0.87
miR-331	1.29	0.70	**miR-374**	1.39	1.71	**miR-99b**	N.D.	0.88
miR-30b	1.34	0.78	**miR-195**	0.62	0.97	**miR-28-3p**	N.D.	0.96
miR-323-3p	2.21	1.73	**miR-223**	0.38	0.74	**miR-376c**	N.D.	0.97
miR-142-3p	1.46	1.04	**miR-20b**	0.23	0.82	**let-7b**	N.D.	1.12
miR-335	0.79	0.39	**miR-24**	0.62	1.23	**let-7g**	N.D.	1.34
miR-145	0.63	0.27	**miR-574-3p**	0.35	0.98	**miR-29c**	N.D.	1.39
miR-221	1.22	0.95	**miR-146b**	0.63	1.41	**miR-26b**	N.D.	1.45
miR-150	2.30	2.05	**let-7d**	0.55	1.42	**miR-155**	N.D.	1.66
miR-20a	1.11	0.87	**miR-186**	0.28	1.26	**miR-494**	N.D.	1.85
miR-30c	1.17	0.94	**miR-16**	0.83	1.82	**miR-152**	N.D.	1.97
miR-15b	0.93	0.70	**miR-27b**	0.91	2.03	**miR-454**	N.D.	2.05
miR-19a	1.17	0.97	**miR-376a**	0.63	1.97	**miR-133a**	N.D.	2.12
miR-17	1.07	0.88	**miR-139-5p**	0.65	2.09	**miR-539**	N.D.	2.37
miR-328	0.93	0.75	**miR-628-5p**	2.32	3.83	**miR-148a**	N.D.	2.85
miR-199a-3p	1.06	0.91	**miR-29a**	0.80	2.40	**miR-518f**	N.D.	2.99
miR-320	1.07	0.93	**miR-122**	0.96	2.97	**miR-627**	N.D.	3.02
miR-191	1.12	0.98	**miR-192**	0.32	3.76	**miR-636**	N.D.	3.73
miR-26a	1.20	1.09	**miR-142-5p**	N.D.	0.35	**miR-301b**	N.D.	3.83
miR-126	1.30	1.20	**miR-526b**	N.D.	0.38	**miR-107**	N.D.	5.30
miR-92a	0.90	0.81	**miR-103**	N.D.	0.45	**miR-410**	N.D.	5.77
miR-197	0.46	0.37	**miR-744**	N.D.	0.45	**miR-424**	N.D.	6.91
miR-484	0.75	0.71	**miR-340**	N.D.	0.48	**miR-487b**	N.D.	6.92
miR-106a	0.92	0.90	**let-7a**	N.D.	0.50	**let-7c**	N.D.	13.26
let-7e	1.53	1.58	**miR-130b**	N.D.	0.56	**miR-486**	0.61	N.D.
miR-222	0.67	0.87	**miR-130a**	N.D.	0.58	**miR-532**	1.11	N.D.
miR-27a	1.38	1.59	**miR-301**	N.D.	0.65	**miR-375**	1.20	N.D.
miR-21	0.66	0.90	**miR-128a**	N.D.	0.72	**miR-423-5p**	1.24	N.D.
miR-342-3p	0.78	1.04	**miR-28**	N.D.	0.75	**miR-212**	1.63	N.D.
miR-146a	0.77	1.03	**miR-345**	N.D.	0.82			
miR-24	4.86	0.92	**miR-99b**	0.82	0.84	**miR-483-5p**	0.43	N.D.
miR-574-3p	3.59	0.86	**miR-146a**	0.58	0.65	**miR-192**	0.59	N.D.
miR-222	3.29	1.20	**miR-146b**	0.55	0.65	**miR-99a**	2.80	N.D.
miR-27b	3.16	1.30	**miR-425-5p**	0.56	0.66	**miR-660**	3.94	N.D.
miR-29c	2.36	0.52	**miR-328**	0.44	0.54	**miR-193a-5p**	6.52	N.D.
miR-152	1.84	0.50	**miR-454**	0.23	0.34	**miR-125b**	16.31	N.D.
miR-376c	2.05	0.73	**miR-150**	0.57	0.71	**miR-193b**	17.65	N.D.
miR-744	2.98	1.81	**miR-323-3p**	0.59	0.78	**miR-200c**	90.15	N.D.
miR-181a	1.67	0.61	**miR-335**	0.26	0.45	**miR-203**	1626.14	N.D.
miR-29a	2.90	2.03	**miR-26b**	1.03	1.24	**miR-532**	N.D.	0.39
miR-221	1.61	0.86	**miR-191**	0.46	0.69	**miR-370**	N.D.	0.47
miR-331	1.67	0.92	**miR-30b**	0.72	0.96	**miR-339-3p**	N.D.	0.52
let-7b	1.30	0.68	**miR-320**	0.58	0.83	**miR-301**	N.D.	0.54
miR-19a	1.17	0.56	**miR-199a-3p**	0.24	0.57	**miR-125a-5p**	N.D.	0.54
miR-636	0.87	0.37	**miR-628-5p**	0.29	0.65	**miR-148b**	N.D.	0.55
miR-27a	1.20	0.72	**miR-223**	0.27	0.63	**miR-590-5p**	N.D.	0.58
miR-106a	1.09	0.64	**miR-30c**	0.59	0.98	**miR-142-5p**	N.D.	0.62
let-7d	1.38	0.99	**miR-374**	0.45	0.83	**miR-597**	N.D.	0.64
miR-20a	0.94	0.55	**miR-126**	0.34	0.73	**miR-133a**	N.D.	0.65
miR-19b	1.09	0.71	**miR-142-3p**	0.30	0.69	**miR-539**	N.D.	0.70
miR-21	0.91	0.71	**miR-28-3p**	0.52	0.93	**let-7a**	N.D.	0.73
miR-423-5p	0.36	0.23	**miR-526b**	0.57	1.05	**miR-127**	N.D.	0.79
miR-197	0.95	0.85	**miR-106b**	0.52	1.03	**miR-18a**	N.D.	0.87
miR-186	0.60	0.51	**miR-139-5p**	0.22	0.76	**miR-411**	N.D.	0.92
let-7g	0.68	0.60	**miR-92a**	0.32	0.88	**miR-324-3p**	N.D.	1.10
miR-20b	0.85	0.78	**miR-484**	0.34	1.15	**miR-185**	N.D.	1.28
miR-652	0.53	0.46	**miR-25**	0.75	1.58	**miR-148a**	N.D.	1.31
miR-26a	0.93	0.87	**miR-103**	0.63	1.46	**miR-130b**	N.D.	1.35
let-7e	0.83	0.78	**miR-145**	0.20	1.11	**miR-155**	N.D.	1.65
miR-16	0.39	0.35	**miR-195**	0.25	1.20	**miR-132**	N.D.	2.02
miR-130a	1.07	1.04	**miR-486**	0.18	1.28	**miR-487b**	N.D.	2.11
miR-342-3p	1.37	1.35	**miR-15b**	0.23	1.35	**miR-28**	N.D.	2.22
miR-17	0.73	0.71	**miR-122**	0.23	9.61			

Arrangement of data is equal to the clockwise order shown in Figure 1; for miRNA data with only one available value (not shown in Figure 1), the miRNAs are arranged in an ascending order.
